# Early weaning from invasive mechanical ventilation via high-flow nasal oxygen *versus* conventional weaning in patients with hypoxemic respiratory failure: a prospective randomized controlled study

**DOI:** 10.62675/2965-2774.20250157

**Published:** 2025-01-15

**Authors:** Hareesh Ayyawar, Pradeep Bhatia, Sadik Mohammed, Nikhil Kothari, Bharat Paliwal, Ankur Sharma

**Affiliations:** 1 Department of Critical Care Medicine Yashoda Group of Hospital Hyderabad India Department of Critical Care Medicine, Yashoda Group of Hospital - Hyderabad, India.; 2 Anaesthesiology and Critical Care All India Institute of Medical Sciences Jodhpur India Anaesthesiology and Critical Care, All India Institute of Medical Sciences - Jodhpur, India.

**Keywords:** Hypoxia, Respiratory insufficiency, Airway extubation, Oxygen inhalational therapy, Respiration, artificial, Pneumonia, ventilator-associated, Intensive care units

## Abstract

**Objective:**

Although the efficacy of high-flow nasal oxygen therapy in delaying or avoiding intubation in patients with hypoxemic respiratory failure has been studied, its potential for facilitating early weaning from invasive mechanical ventilation remains unexplored.

**Methods:**

In this randomized controlled trial, 80 adults with acute hypoxemic respiratory failure requiring invasive mechanical ventilation for > 48 hours were enrolled and divided into two groups: conventional weaning and early weaning via high-flow nasal oxygen. In the conventional weaning group, the spontaneous breathing trial was performed after the PaO_2_/FiO_2_ ratio was ≥ 200, whereas in the high-flow nasal oxygen group, the spontaneous breathing trial was conducted earlier when the PaO_2_/FiO_2_ ratio was 150 - 200. Following each successful spontaneous breathing trial, patients were extubated and put on oxygen supplementation via a venturi mask or high-flow nasal oxygen on the basis of their group allocation. The primary objective was to compare extubation failure (reintubation within 48 hours). The secondary objectives were to compare total invasive mechanical ventilation, oxygen requirement and sedation requirement days, ventilator-associated pneumonia incidence, invasive mechanical ventilation-free days, intensive care unit length of stay, and intensive care unit all-cause mortality.

**Results:**

Extubation failure was not significantly different between the high-flow nasal oxygen group and the conventional weaning group [12.5% *versus* 25%, respectively; odds ratio (95%CI) 0.5 (0.19 - 1.33)] (p = 0.25). Early weaning from invasive mechanical ventilation via high-flow nasal oxygen was associated with significantly increased invasive mechanical ventilation-free days and total oxygen requirement days (p = 0.02 and p = 0.01, respectively). No significant between-group differences were observed in total invasive mechanical ventilation days, ventilator-associated pneumonia incidence, intensive care unit length of stay, sedation duration, or all-cause mortality.

**Conclusion:**

Among patients with acute hypoxemic respiratory failure, early extubation with high-flow nasal oxygen is a feasible and superior alternative to the conventional method of weaning, as it increases the number of invasive mechanical ventilation-free days.

## INTRODUCTION

The requirement of invasive mechanical ventilation (IMV) is one of the main indications for intensive care unit (ICU) admission. Although it is a lifesaving therapy, its prolonged duration has been found to be associated with increased morbidity and mortality.^1,2^Successful and early weaning from IMV is important to improve outcomes in critically ill ICU patients.^1^

High-flow nasal oxygen (HFNO) therapy allows high flows and fractions of inspired oxygen (FiO_2_) at more physiological temperatures and humidity levels. Currently, it is increasingly used in ICU and emergency department settings to manage patients with acute hypoxemic respiratory failure (ARF) and optimize preoxygenation before intubation in patients with mild-to-moderate hypoxemia. The mechanisms through which HFNO acts include small pliable nasal prongs that increase comfort, warming and humidification of secretions that facilitate expectoration, washout of nasopharyngeal dead space that improves ventilation efficiency, and high flow rates that aid in the reliable delivery of FiO_2_ and a small positive end-expiratory pressure (PEEP) effect.

The application of HFNO has been studied extensively by comparing it with conventional oxygen therapy (COT) and noninvasive ventilation (NIV) in the prevention of IMV in patients with ARF;^3-7^its applications have also been examined as postextubation oxygen therapy in high-risk patients compared with COT and NIV.^8-12^Compared with COT, among extubated patients at low risk for reintubation, HFNO therapy also showed good results in reducing the risk of reintubation.^9^ The application of NIV after early extubation has been studied in patients with hypoxemic and hypercapnic respiratory failure.^13,14^ In most of the literature, conventional weaning criteria were followed, and patients were extubated after achieving a PaO_2_/FiO_2_ (P/F) ratio of > 200, an FiO_2_ of < 0.4 and a PEEP of 5 - 8cmH_2_O. However, there are currently no data on the use of HFNO to facilitate the process of early weaning (extubation at a P/F ratio of = 150 - 200 with an FiO_2_ of 0.5 and a PEEP of 8cmH_2_O) from IMV compared with the conventional weaning method in patients with ARF.

Therefore, we designed a study to assess the feasibility of early weaning from IMV followed by immediate HFNO compared with conventional weaning in patients with resolving ARF. We hypothesized that early weaning from IMV via HFNO would be a feasible and better alternative to the conventional weaning method. The primary objective of the study was to compare the incidences of extubation failure. The secondary objectives were to compare the total IMV days, ventilator-associated pneumonia (VAP) incidence, IMV-free days, total oxygen requirement days, days of sedation requirement, ICU length of stay, and all-cause mortality during the ICU stay.

### Study design and methods

This present prospective, randomized controlled trial was conducted in the Department of Anaesthesiology and Critical Care at the All India Institute of Medical Sciences (AIIMS), Jodhpur. After approval from the institutional ethical committee (certificate reference number: AIIMS/IEC/2021/3313 dated 12/03/2021), the study was registered prospectively with the clinical trial registry of India (CTRI: ctri.nic.in) (CTRI/2021/07/034659 dated 07/07/2021; recruitment period July 2021 to June 2022). All the procedures followed were in accordance with the ethical standards of the local institutional committee on human experimentation and the Helsinki Declaration. Adult patients aged over 18 years who were admitted to the ICU and received IMV through an endotracheal tube for more than 48 hours due to ARF were enrolled after written informed consent was provided by the patient or his or her first-degree blood relative. Patients with altered sensorium or profound neurological deficits or myopathy; patients with any of the contraindications for HFNO application (base of skull fracture, known nasal obstruction, or nasal trauma); psychiatric, agitated, or noncooperative patients; obese patients; patients with chronic lung disease or hypercapnic respiratory failure; and pregnant patients were excluded.

Randomization was performed via a computer-generated random number table, and patients were allocated into two groups: the conventional group (patients who received SBT with pressure support of 5 - 8cmH_2_O, a PEEP of 5cmH_2_O and a FiO_2_ of ≤ 0.4 after achieving a P/F ratio of ≥ 200 with FiO2 of ≤ 0.4) and the HFNO group (patients who received SBT with pressure support of 8cmH_2_O with the same PEEP and FiO_2_ at a P/F ratio of 150 - 200 with FiO_2_ of = 0.5 and a PEEP of 8cmH_2_O). Allocation concealment was achieved using sealed opaque envelopes that were opened just after patient enrollment. Due to the intervention that was selected, blinding was not possible.

All patients received appropriate continuous sedation titrated to a Richmond Agitation Sedation Scale (RAAS) score^15^ of −2 to 0. After recruitment, all patients were screened daily for their readiness to wean on the basis of the following criteria;^16,17^awake and alert mentally or easily arousable, improvement in the cause of the respiratory failure, adequate cough reflex, ability to initiate an inspiratory effort with a trigger flow of 2L/minute, Hb ≥ 7gm/dL, pH > 7.25, core body temperature ≤ 37.5°C, hemodynamic stability with no or minimal inotropic support, and rapid shallow breathing index (RSBI) (respiration rate/tidal volume) < 105. In both groups, the PEEP and FiO_2_ were adjusted by the treating physician as per the requirement of patients. Following a decision by the treating clinician based on the above criteria, all patients underwent SBT in alignment with the group to which they were randomly assigned.

During the SBT, the patients’ vital signs and ventilator parameters, such as tidal volume and respiratory rate (minute ventilation), were monitored. In addition, their respiratory distress and mental status changes, alertness, and responsiveness were assessed. At the end of the 30-minute SBT, an arterial blood gas (ABG) sample was obtained for each patient. SBT success was defined as maintenance of the same P/F ratio (≥ 200 in the conventional group and ≥ 150 - 200 in the HFNO group) and an RSBI of < 105 with acceptable clinical parameters. In the case of failed SBT, no further SBT was performed within the next 24 hours.

After successful SBT, the patients were extubated and put on oxygen supplementation on the basis of their group assignment. Those in the HFNO group received oxygen supplementation through HFNO with a 60L flow and a 1.0 FiO_2_ and were subsequently titrated to maintain an RR ≤ 30 and SpO_2_ of ≥ 94%. In contrast, the patients in the conventional group were placed on oxygen supplementation via a venturi mask with an FiO_2_ of 1.0 and were titrated to maintain an SpO_2_ of ≥ 94% and an RR ≤ 30.

All the patients’ vital signs, SpO_2_, electrocardiogram, RR, and signs of distress, such as accessory muscle use, tachypnea, tachycardia, chest retractions, agitation, and mental state changes, were monitored in the postextubation period. In the case of extubation failure based on the clinical and ABG parameters as assessed by the treating clinician, patients were reintubated again and put on IMV. Reintubation within 48 hours after extubation was classified as extubation failure, and these patients received further IMV and weaning via the conventional weaning method.

### Statistical analysis

The sample size was calculated on the basis of a previous study^10^ (Song et al.), where the outcome (failure) proportions between the two groups (receiving either HFNO or COT postextubation) were 0.10 and 0.367, respectively. With a confidence interval of 95% (95%CI) and power of 80%, the sample size required to detect this difference was 38 in each group.

The data were recorded in a Microsoft Excel spreadsheet. Statistical analysis was conducted using the Statistical Package for Social Sciences version 23 (IBM SPSS Statistics for Windows, Version 23.0; Armonk, NY: IBM Corp., NY, USA) for Windows. Nominal variables are presented as counts or percentages and were analyzed via the chi-square test. Ordinal variables and continuous variables with nonnormal distributions are presented as medians and quartiles and were analyzed via the Mann–Whitney U test for unpaired data and the Wilcoxon rank sum test for paired data. Continuous variables with a normal distribution are presented as means ± standard deviations (SDs) and were analyzed via unpaired t tests and paired t tests for independent and dependent samples. The risk of extubation failure is presented as the odds ratio (95%CI). Significance was defined as a two-sided p value less than 0.05.

## RESULTS

**Figure 1:**
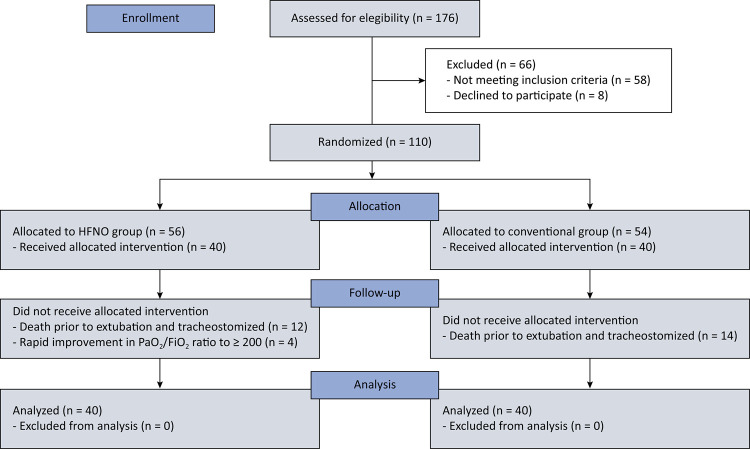
Consort flow diagram.

During the study period, 176 patients with ARF received IMV for more than 48 hours. Of them, 66 were excluded (58 did not meet the inclusion criteria, 8 declined to participate). Of the remaining 110 patients, 56 were included in the HFNO group, and 54 were included in the conventional group. After enrollment, 12 patients in the HFNO group and 14 patients in the conventional group could not receive the allocated treatment due to mortality or tracheostomy during the course of treatment. Additionally, 4 patients in the HFNO group improved rapidly (P/F ratio > 200). Eventually, 40 patients in each group were treated, and their data were analyzed (Figure 1). Both groups were comparable with respect to their demographic characteristics (age, gender), diagnostic category (cause of acute respiratory distress syndrome [pulmonary/extrapulmonary]), comorbidities (diabetes, hypertension, hypothyroidism, etc.), duration of illness before ICU admission, P/F ratio at admission, ICU admission scores (Sequential Organ Failure Assessment [SOFA] and Acute Physiology and Chronic Health Evaluation II [APACHE II]) and number of SBTs required before extubation (p > 0.05) (Table 1). The PaO_2_ did not differ between the groups, whereas the FiO_2_ differed significantly before and after SBT. In accordance with the study protocol, the P/F ratios before SBT, after SBT, and after extubation were compared between and within the groups. There was a significant decrease in the P/F ratio after both SBT and extubation compared with before SBT in both groups (p < 0.001). The patients in the HFNO group were extubated at a significantly lower P/F ratio than those in the conventional group were (p < 0.001) (Table 2). The RSBI scores were comparable between the two groups both before and after SBT (p > 0.05) (Table 2).

HFNO - high-flow nasal oxygen; PaO_2_/FiO_2_ - partial pressure of arterial oxygen/fraction of inspired oxygen ratio.

**Table 1:** Comparisons of baseline characteristics (age, gender), diagnostic categories, comorbidities, durations of illness and intensive care unit admission scores between the groups

	HFNO (n = 40)	Conventional (n = 40)	Median diff (95%CI)	p value
Age (years)	48 (30 - 61)	50.5 (32 - 64)	-2.5 (-2.1 - 2.2)	0.2
Gender^b^ (M/F) (%)	25/15 (62.5/37.5)	28/12 (70/30)	-	0.63
Comorbidities (Y/N) (%)	22/18 (55/45)	24/16 (60/40)	-	0.12
Diagnostic category				
Pulmonary/extrapulmonary (%)	34/6 (85/15)	31/9 (77.5/22/5)	-	0.57
Duration of illness (days)	6 (3 - 7)	5 (3-9)	1 (-1.4 - 1. 6)	0.61
SOFA score at admission	7 (4 - 7)	6 (5-9)	1 (-2.7 - 0.23)	0.06
APACHE II score at admission	14 (9 - 18)	15 (9 - 19)	-1 (-5.1 - 0.5)	0.13
PaO_2_/FiO_2_ at admission	122 (110 - 130)	135 (105 - 160)	-13 (-25.0 - 3.4)	0.06
Number of SBTs before extubation	1 (1 - 1)	1 (1 - 1)	0 (-0.9 - 0.2)	0.08

HFNO - high-flow nasal oxygen; 95%CI - 95% confidence interval; M - male; F - female; Y - yes; N - no; SOFA - Sequential Organ Failure Assessment; APACHE II - Acute Physiology and Chronic Health Evaluation II; PaO_2_/FiO_2_ - partial pressure of arterial oxygen/fraction of inspired oxygen ratio; SBT - spontaneous breathing trial.

The Mann–Whitney test was used to compare age, duration of illness, SOFA score and APACHE II score, whereas the chi–square test was used to compare gender, comorbidities and diagnostic categories between the groups. Data are presented as medians (quartiles) or numbers.

**Table 2:** Comparisons of PaO2, FiO2, P/F ratio, rapid shallow breathing index and positive end-expiratory pressure at different time points between the groups

	HFNO (n = 40)	Conventional (n = 40)	Median diff (95%CI); p value
PaO_2_			
Prior to SBT	89.5 (65 - 98)	96 (68 - 118)	6.5 (-15.5 - 1.2); 0.08
After SBT	78.5 (59 - 94)	87.5 (65 - 110)	9.5 (-18 - 0.2); 0.054
FiO_2_			
Prior to SBT	0.55 (0.5 - 0.6)	0.35 (0.3 - 0.4)	0.2 (-0.3 - -0.13); 0.001
After SBT	0.5 (0.5 - 0.55)	0.35 (0.35 - 0.4)	0.15 (-0.26 - -0.11); 0.001
PaO_2_/FiO_2_			
Prior to SBT	183 (166 - 192)	280 (260 - 318)	-97 (-129 - -23); 0.001
After SBT	175 (160 - 186)	265 (230 - 290)	-90 (-112 - -73); 0.001
Postextubation	173 (160 - 184)	258 (236 - 286)	-85 (-115 - -77); 0.001
p value	0.001	0.001	-
RSBI			
Before SBT	75.5 (65 - 80)	71.5 (54 - 80)	4.0 (-2.7 - 9.7); 0.24
After SBT	76 (68 - 86)	79 (59 - 89)	3.0 (-4.3 - 8.3); 0.67
Median diff (95%CI); p value	0.5 (-4.7 - -1.5); 0.001	7.7 (-5.9 - -3.4); 0.001	`
PEEP at the time of extubation	7 (6 - 8)	5 (5 - 6)	2 (-3.5 - -1.5); 0.001

95%CI - 95% confidence interval; PaO_2_ - partial pressure of arterial oxygen; SBT - spontaneous breathing trial; FiO_2_ - fraction of inspired oxygen; PaO_2_/FiO_2_ - partial pressure of arterial oxygen/fraction of inspired oxygen ratio; RSBI - rapid shallow breathing index; PEEP - positive end-expiratory pressure.

The Mann–Whitney test was used to compare the data between the groups. The Friedman test was used to compare the partial pressure of arterial oxygen/fraction of inspired oxygen ratio within each group, whereas the Wilcoxon signed-rank test was used to compare the rapid shallow breathing index within each group. Data are presented as the medians (quartiles).

**Figure 2:**
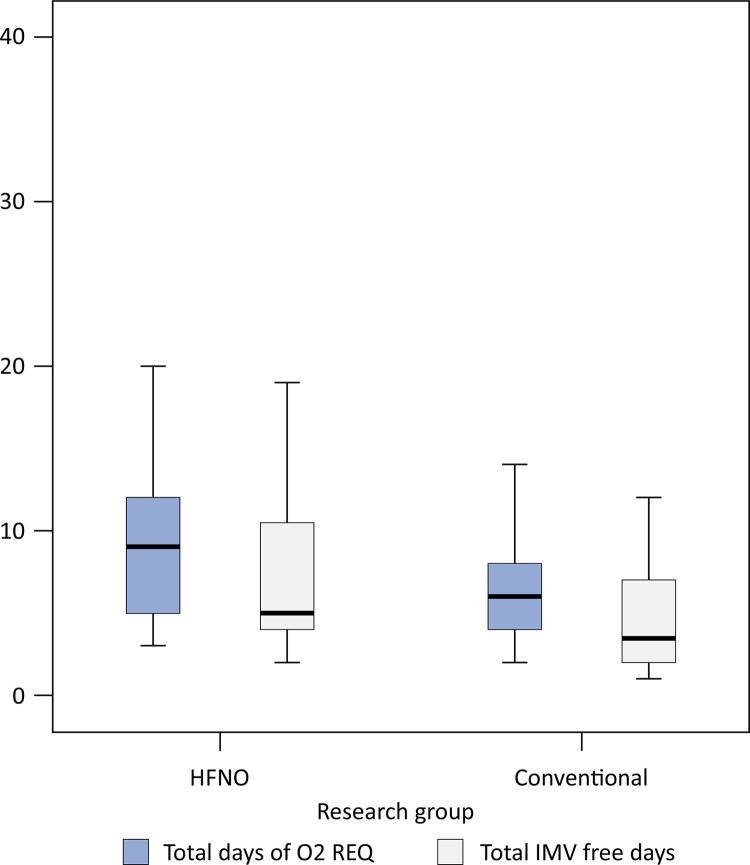
Box and whisker plots comparing the median (quartiles) total oxygen requirement days and total invasive mechanical ventilation-free days between the high flow nasal oxygen and conventional weaning techniques.

Extubation failure was observed in 5 patients (12.5%) in the HFNO group and 10 patients (25%) in the conventional group (Table 3). The risk of extubation failure did not significantly differ between the HFNO group and the conventional weaning group [odds ratio (95%CI) 0.5 (0.19 - 1.33)] (p = 0.25). As a consequence of early weaning, the HFNO group had significantly more total IMV-free days (days between extubation and ICU discharge/death) [median difference (95% CI) 1 (1.8 - 4.1)] (Figure 2) and total oxygen requirement days [median difference (95% CI) 3 (1.3 - 4.4)] than the conventional group did (p = 0.02 and p = 0.01, respectively) (Figure 2).

**Table 3:** Comparisons of other outcomes between the groups

	HFNO (n = 40)	Conventional (n = 40)	Median diff (95%CI)	p value
Failed weaning*	5 (12.5)	10 (25)	-	0.25
Total IMV days† before extubation	4 (3 - 6)	4 (3 - 6)	0 (-1.6 - 1.2)	0.54
Total sedation requirement days†	5 (3 - 7)	5 (3 - 6)	0 (-0.63 - 2.5)	0.27
Total ICU length of stay†	11 (7 - 15)	8 (6 - 12)	3 (-1.9 - 4.7)	0.07
VAP rate*	8 (20)	5 (12.5)	-	0.55
Reintubation after 48 h of extubation*	1 (2.5)	3 (7.5)	-	0.62
All-cause mortality*	2 (5)	5 (12.5)	-	0.43

VAP - ventilator-associated pneumonia; 95%CI - 95% confidence interval; IMV - invasive mechanical ventilation; ICU - intensive care unit. * Data compared via the chi-square test; † Data compared via the Mann–Whitney test. Data are presented as numbers (%) or medians (quartiles).

IMV - invasive mechanical ventilation; HFNO - high-flow nasal oxygen.

There was no significant difference in the other outcome measures of total IMV days, VAP rates, days of sedation requirement, or ICU length of stay between the groups (p > 0.05) (Table 3). Reintubation after 48 hours was observed in 2.5% of patients in the HFNO group and 7.5% of patients in the conventional group (p = 0.615) (Table 3). The risk of all-cause mortality did not differ significantly between the HFNO and conventional groups [odds ratio (95%CI) 0.37 (0.07 - 2.02)] (Table 3).

## DISCUSSION

The present study demonstrated that in critically ill patients with resolving ARF, early weaning from IMV via HFNO is a feasible and better alternative to conventional weaning, as it significantly increases the number of IMV-free days without increasing the risk of extubation failure. Additionally, it did not adversely affect total IMV days, VAP rates, days of sedation requirement, ICU length of stay, or all-cause mortality during the ICU stay.

Invasive mechanical ventilation is an essential part of management for most critically ill patients who are admitted to the ICU. Its prolonged use has been shown to increase morbidity and mortality, and successful early weaning from IMV plays a crucial role in improved outcomes.^1,2^The role of NIV in early weaning from IMV has been investigated in both hypercapnic and hypoxemic respiratory failure, in which the use of NIV was found to be associated with significantly more IMV-free days; in addition, it was found to decrease the number of IMV days and total ventilator days.^13,^^14^However, similar data relating to the use of HFNO as a means of facilitating the process of early weaning from IMV in ARF patients are lacking. Therefore, our study explored the role of HFNO in successful early weaning, i.e., after a P/F ratio of 150 - 200 is achieved, compared with the conventional method, which involves a P/F ratio ≥ 200.

By delivering high flows (up to 60L/minute) of a warm and humidified oxygen–air mixture through nasal prongs, HFNO generates mild positive pressure. It may prevent extubation failure after early extubation via mechanisms, such as controlling oxygen, reducing hypoxia, minimizing CO_2_ rebreathing, lowering the respiratory rate, and improving gas exchange.^18,19^A minimal PEEP improves patient comfort by preventing lung collapse, reducing airway inflammation, and enhancing secretion drainage.^18,20^ In a study by Hernández et al.,^9^ postextubation HFNO was compared with COT in low-risk patients with a P/F ratio of ≥ 150, resulting in reduced reintubation risk within 72 hours. They predefined the extubation criterion as a P/F ratio ≥ 150; however, the mean P/F ratios before extubation were 227 ± 25 in the HFNO group and 237 ± 34 in the conventional group. Our study adhered to strict oxygenation criteria: 150 - 200 for HFNO and ≥ 200 for conventional weaning.

Our study demonstrated that early weaning from IMV via HFNO is as effective as conventional weaning in critically ill patients with ARF. The reintubation rate in the HFNO group (12.5%) aligned with those published in previous reports.^21^ Within 48 hours, we observed lower extubation failure in the HFNO group (12.5%) than in the conventional group (25%), although the difference was not statistically significant. In a study by Song et al., the effectiveness of HFNO therapy was compared with that of conventional oxygen (administered via an air entrainment mask) postextubation in patients with ARF.^10^ They reported a significantly higher success rate with HFNO than with conventional oxygen (90% *versus* 63.3%, respectively). In their study, all the patients were extubated on achieving a P/F ratio of ≥ 150 with a FiO_2_ of ≤ 0.4 and PEEP ≤ 8cmH2O, which might have led to greater failure with conventional oxygen. To reduce the failure rates in the conventional group, we extubated the patients after they achieved a P/F ratio of ≥ 200 and a P/F ratio of 150 - 200 in the HFNO group. The benefit of successful early weaning translated into more IMV-free days in the HFNO group.

In our study, the observed reintubation rate was lower in the HFNO group than in the conventional group (2.5% *versus* 7.5%, respectively), which is in line with rates in the published literature on similar topics.^22-25^Maggiore et al. demonstrated the benefits of HFNO in critically ill patients, showing improved oxygenation and comfort and a lower reintubation rate (3.8%).^19^ Hernández et al. reported that HFNO lowered the reintubation rate (4.9%) compared with COT (12.2%) in low-risk patients.^9^ This rate is influenced by various ICU factors and is often not reported in clinical trials. The optimal duration of HFNO remains an area of uncertainty, but in our study, it was continued until patients were ready to transition to low-flow oxygen therapy. Ventilator-associated pneumonia occurred in 20% of patients in the HFNO group and 12.5% of patients in the conventional group. Despite the potential impact of increased IMV-free days on reducing VAP, our study did not uncover such a difference, likely due to similar total IMV days and the relatively small sample size. Hernández et al.^9^ found no difference in ICU length of stay despite the lower reintubation rate in the HFNO group than in the conventional group. These findings align with those of our study, where there were no significant differences between the groups in terms of total IMV days or ICU length of stay. Although the HFNO group underwent early extubation, no significant differences in total mean IMV days were observed between the groups, possibly due to the lower admission P/F ratio in the HFNO group than in the conventional weaning group.

In our study, the risk of all-cause mortality was similar between the HFNO and conventional weaning techniques. Hernández et al. also reported no difference in ICU mortality between HFNO and conventional oxygen therapy, supporting our conclusion that early extubation on HFNO does not impact mortality compared with the conventional method.^9^

A few limitations of our study should be kept in mind when interpreting the results. First, patients were extubated only after passing the SBT in both groups, which might call into question “early weaning”; therefore, further studies with patients who did not pass an SBT or still had a high PEEP and/or PS and FiO_2_ to be challenged with an SBT are needed. Second, data regarding ARF etiology and type of comorbidity, which might have influenced weaning outcomes, were not collected or compared. Third, it was not possible to employ blinding; hence, the associated bias could not be eliminated. Fourth, the single-center trial design limited the generalizability of the results. Fifth, the calculated sample size might have been small, as it was based on data from a previous study in which both groups were extubated after achieving similar P/F ratios. Therefore, additional studies with multicenter designs and larger sample sizes are warranted to confirm the results of our study.

## CONCLUSION

In patients with resolving acute hypoxemic respiratory failure, early extubation with high flow nasal oxygen is feasible and provides a better alternative to conventional weaning from invasive mechanical ventilation, as it is associated with more invasive mechanical ventilation free days without adversely affecting other outcomes.
